# The Caregiver Burden Questionnaire for Heart Failure (CBQ-HF): face and content validity

**DOI:** 10.1186/1477-7525-11-84

**Published:** 2013-05-25

**Authors:** Louise Humphrey, Karoly Kulich, Celine Deschaseaux, Steven Blackburn, Laura Maguire, Anna Strömberg

**Affiliations:** 1 Adelphi Values, Adelphi Mill, Bollington, Macclesfield, Cheshire, SK10 5JB, UK; 2 Novartis Pharma AG, Basel, Switzerland; 3 Division of Nursing Science, Department of Medical and Health Sciences, Linköping University, Linköping, Sweden; 4 Department of Cardiology, County Council of Östergötland, Linköping, Sweden

**Keywords:** Heart failure, Caregiver burden, Instrument development, Patient-reported outcome, Content validity, Face validity, Family

## Abstract

**Background:**

A new caregiver burden questionnaire for heart failure (CBQ-HF v1.0) was developed based on previously conducted qualitative interviews with HF caregivers and with input from HF clinical experts. Version 1.0 of the CBQ-HF included 41 items measuring the burden associated with caregiving in the following domains: physical, emotional/psychological, social, and impact on caregiver’s life. Following initial development, the next stage was to evaluate caregivers’ understanding of the questionnaire items and their conceptual relevance.

**Methods:**

To evaluate the face and content validity of the new questionnaire, cognitive interviews were conducted with caregivers of heart failure patients. The cognitive interviews included a “think aloud” exercise as the patient completed the CBQ-HF, followed by more specific probing questions to better understand caregivers’ understanding, interpretation and the relevance of the instructions, items, response scales and recall period.

**Results:**

Eighteen caregivers of heart failure patients were recruited. The mean age of the caregivers was 50 years (SD = 10.2). Eighty-three percent of caregivers were female and most commonly the patient was either a spouse (44%) or a parent (28%). Among the patients 55% were NYHA Class 2 and 45% were NYHA Class 3 or 4. The caregiver cognitive interviews demonstrated that the CBQ-HF was well understood, relevant and consistently interpreted. From the initial 41 item questionnaire, fifteen items were deleted due to conceptual overlap and/or item redundancy. The final 26-item CBQ-HF (v3.0) uses a 5-point Likert severity scale, assessing 4 domains of physical, emotional/psychological, social and lifestyle burdens using a 4-week recall period.

**Conclusions:**

The CBQ-HF (v3.0) is a comprehensive and relevant measure of subjective caregiver burden with strong content validity. This study has established that the CBQ-HF (v3.0) has strong face and content validity and should be valuable as an outcomes measure to help understand and monitor the relationship between patient heart failure severity and caregiver burden. A Translatability Assessment^SM^ of the measure has since been performed confirming the cultural appropriateness of the measure and psychometric validation is planned for the future to further explore the reliability, and validity of the new questionnaire in a larger caregiver sample.

## Background

Chronic Heart Failure (HF) is a complex clinical syndrome in which patients have symptoms (e.g. breathlessness, ankle swelling, fatigue) and signs (e.g. elevated jugular venous pressure, pulmonary crackles, and displaced apex beat) as a result of the heart’s inability to supply sufficient blood flow to meet the body’s needs [1,2]. HF affects 1–2% of the adult population in developed countries, rising to ≥10% among those aged >80 years [1]. Prior to 1990, the prognosis for patients with severe HF was poor with up to 60% dying within five years of diagnosis; however conventional HF treatment has since significantly decreased mortality and hospitalization rates [1]. Quality of life in HF patients is significantly impaired, predominantly as a result of the physical limitations imposed by the disease which can also lead to social limitations and emotional problems [3,4]. In adjusting to the impact of the symptoms associated with the disease, patients with HF can become increasingly dependent on caregivers [5].

The aim of this study was to evaluate the face and content validity of a new HF caregiver burden measure (the Caregiver Burden Questionnaire Heart Failure – CBQ-HF). A caregiver has been defined as an adult, other than the person’s general practitioner, specialist physician or other health care professional, who has significant responsibility for managing the well-being of a person diagnosed with a chronic or debilitating medical condition [6]. Typically unpaid or uncompensated, caregivers provide assistance to the patient in the day to day living activities, particularly where the disease is severe and associated with considerable functional impairment [7]. The importance of effective caregivers on HF patient outcomes has been shown in improved quality of life [8], lower hospitalization rates [9,10] and reduced mortality [9-11]. However, providing regular care to HF patients can lead to deficits in caregiver’s own health and quality of life [12-14]. Studies have shown that HF caregivers experience similar levels of burden as caregivers of patients with advanced cancer and chronic obstructive pulmonary disorder [15,16]. Moreover, the measurement of caregiver burden is becoming increasingly important, as the role of caregivers in supporting individuals with chronic illnesses such as HF is increasingly recognised by wider society and the scientific community [17].

In understanding the nature and impact of caregiver burden in HF, it is essential to have robust and appropriate instruments for measurement and evaluation [18,19]. As part of an initial targeted literature review, a number of existing measures were identified that assess caregiver burden in HF, including two disease-specific instruments such as FAMQOL [20] and the Dutch Objective Burden Inventory (DOBI) [21,22] as well as more general scales developed for both chronic physical and mental impairments such as the Caregiver Reaction Assessment [23] and the Zarit Burden Inventory [24]. From this review, and previous qualitative research conducted with HF caregivers [25], a conceptual model was developed. The review concluded that while existing instruments address multiple aspects of caregiver burden, there is a lack of evidence of content validity among HF caregivers, with most not involving qualitative input from HF caregivers during their development, and/or a lack of conceptual coverage to measure all the relevant burden concepts for HF caregivers included in the conceptual model. Content validity is a key consideration for regulatory approval of a Clinical Outcome Assessments (COA). It is defined as the extent to which the construct of interest is comprehensively sampled by the domain and items in the scale [26]. This is particularly important when measuring a subjective concept such as burden i.e. a measure of caregiver burden should reflect what is important to the target population (in this case, HF caregivers) and be comprehensive in covering their concerns [18,19]. Qualitative input in both item generation and evaluation of understanding are recommended methods to assess content validity of COAs [18,19]. Qualitative input from the target population is also important to ensure the questionnaire has sufficient face validity; with consideration to the low levels of health literacy observed in many caregivers [27], the instructions and questions must use language used by caregivers themselves to describe their experiences of burden (i.e. simple, culturally appropriate and free of medical or scientific jargon) [28]. Since existing measures failed to meet these requirements, a new instrument was needed to measure HF caregiver burden that meets the regulatory standards for COAs to be used in clinical trial settings [18,19]).

Thus research was conducted prior to the current study to develop the CBQ-HF. Items for version 1.0 of the CBQ-HF were generated based on the conceptual model and previous qualitative research with HF caregivers [25]. The CBQ-HF assessed four burden domains: 1) Physical burdens; 2) Psychological or Emotional burdens; 3) Social burdens; and 4) Lifestyle burdens. The CBQ-HF, designed as a paper and pen questionnaire, initially contained 41 items each beginning with a common stem (‘How much has caregiving…’). Items were rated on a 5-point Likert scale anchored at ‘not at all’ and ‘a lot’. All items were answered using a recall period of the past four weeks [29].

Following item generation, the next step was to assess the face and content validity of the CBQ-HF. Face validity was based on the caregivers’ understanding and interpretation of the items; content validity was based on the relevance of the items to HF caregivers. This paper presents the results of the validation research.

## Methods

Qualitative interviews were conducted with 18 HF caregivers to evaluate the face and content validity of version 1.0 of the CBQ-HF. This sample size is in keeping with recommended numbers for cognitive interviewing [30].

### Recruitment of participants

Caregivers were identified via HF patients recruited from general practioner clinics in two cities in the United States in 2012: Philadelphia, Pennsylvania, and St Paul, Minnesota. In the first instance, physicians identified eligible patients; then their caregivers were recruited for participation in an interview. To participate in the cognitive interviews, caregivers had to be the primary caregiver of a patient with HF. Targeted sampling methods were used to recruit a representative range of patients and their caregivers and to ensure generalizability of the results. To ensure that the CBQ-HF included concepts that reflect a representative experience [31], caregivers were included from each gender, a range of ages, education levels, ethnicities, work status and patient-caregiver relationships. Including individuals from a range of educational backgrounds was particularly important for testing comprehension of the item wording [32]. Furthermore, the caregivers cared for patients diagnosed with a range of HF severity levels, as measured by New York Heart Association (NYHA) classification. Furthermore, patients with either a reduced ejection fraction (HFrEF) or preserved ejection fraction (HFpEF) were also included.

The study was approved by a US centralised Independent Review Board and conducted in accordance with the Declaration of Helsinki. Written informed consent was obtained from all patients and caregivers prior to entry into the study.

### Interview methods

The methods used in this study were in accordance with the regulatory standards for development and validation of COA measures [18]. All interviews, 90 minutes in duration, were conducted by two trained qualitative interviewers. Following a short open-ended discussion about HF caregiving, caregivers were asked to complete version 1.0 of the CBQ-HF using a ‘think aloud’ exercise – a recommended method for cognitive debriefing [32]. ‘Think-aloud’ involves the respondent completing the questionnaire and speaking aloud their thoughts as they read each instruction and complete each item [33]. This allows access to the participant’s genuine thoughts as they complete the instrument [33]. Following this, caregivers were asked detailed questions about the definitions/meanings, understanding/clarity and relevance of the items, response options and recall period. Specific questions designed to assess the adequacy of concept coverage were also posed. For the concepts ‘physical effort’ and ‘overly relied on’ alternative item versions were presented on showcards to assess caregivers’ preferences.

### Qualitative analysis

All interviews were audio-taped and transcribed verbatim for the purpose of qualitative analysis. Written interview transcripts were then entered into a qualitative software package (Atlas.Ti) (ATLAS.ti Scientific Software Development GmbH, Berlin, Germany) which was used to facilitate analysis of interview transcripts. Interview transcripts were analysed using a thematic approach involving organising quotes into domains and sub-domains as common themes emerged in the data [34]. Content and thematic analysis methods were used to present count and verbatim examples of caregiver responses during the cognitive debriefing interviews [35]. This analysis focused specifically on whether the concepts and items comprising the CBQ-HF were relevant, appropriate and understood by caregivers in the way intended by the developers [18].

### Iterative development of the CBQ-HF

**Figure 1:**
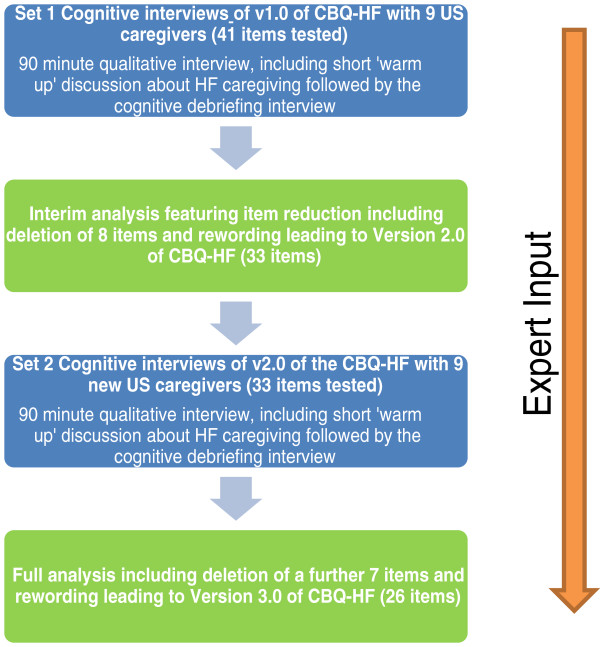
Flow diagram of process of face and content validity testing of CBQ-HF.

Qualitative analysis of the interviews transcripts was conducted in two steps. Firstly, following interviews with the first half of the sample (n = 9) (set 1), data was analyzed to explore whether any changes to the questionnaire or interview guide were required at that point. Version 2.0 of the CBQ-HF was then tested in the last half of the sample (n = 9) (set 2) using the same interview approach as described for the first half. Following the analysis of all 18 cognitive interviews, the final version of the CBQ-HF was agreed and is referred to as version 3.0. A summary of this process is shown in Figure 1 below.

## Results

### Sample characteristics (including caregiver and patient clinical characteristics)

The mean age of the 18 caregivers was 50 years (SD = 10.2). Eighty-three percent of caregivers were female and most commonly the patient was either a spouse (44%) or a parent (28%). All caregivers provided care for at least 8 hours per week with just over half (n = 10) reporting that they provide care “24 hours a day”, “all the time” or lived with the patient thus provided care as required. Fifty percent of the patients were female, 55% were NYHA Class 2 and 45% were NYHA Class 3 or 4. Further details on the caregivers and patients included in the study are presented in Table 1 and Table 2, respectively.

**Table 1:** Demographic characteristics of caregivers

**Caregiver sample characteristics**	**Set 1 St Paul (n = 9)**	**Set 2 Philadelphia (n = 9)**	**Total (N = 18)**
**Age of caregiver**			
Mean (SD)	54 (8.6)	47 (10.6)	50 (10.2)
**Caregiver gender**			
Female	6	9	15
Male	3	0	3
**Caregiver ethnicity**			
African American	1	7	8
Caucasian	6	2	8
Asian	2	0	2
**Relation of person cared for**			
Spouse	5	3	8
Parent	3	2	5
Other^1^	0	4	4
Sibling (brother/sister)	1	0	1
**Gender match of caregiver-patient**			
Gender match (male-male, female-female)	7	5	12
Gender mis-match (male–female, female–male)	2	4	6
**Hours per week typically provide care for (Caregiver reported)**			
8 – 24 hours	2	4	6
25 – 40 hours	1	1	2
41 – 56 hours	0	0	0
57+ hours^2^	6	4	10
**Caregiver living situation**			
Living with the person you care for and other family^3^	3	6	9
Does not live with the patient	2	3	5
Living with the patient ONLY	4	0	4
**Caregiver education status**			
College or university degree (2 or 4 years)	3	5	8
High school diploma or General Educational Diploma (GED)	2	1	3
Some years of college	0	3	3
Certificate program	3	0	3
Graduate or professional	1	0	1
**Caregiver work status**			
Paid work full or part-time	6	8	14
Retired	1	1	2
Not working due to a medical condition	2	0	2

^1^Other includes grandmother, grandfather, godfather and aunt.

^2^ Includes caregivers who reported they provided care for 24 hours per day or “All the time”.

^3^Other family includes partner of caregiver, children of caregiver, and partner of the person cared for.

**Table 2:** Demographic and clinical characteristics of patients

**Patient sample characteristics**	**Set 1 St Paul (n = 9)**	**Set 2 Philadelphia (n = 9)**	**Total (N = 18)**
**Patient gender**			
Male	4	5	9
Female	5	4	9
**Patient NYHA classification**^**4**^			
Class 2: Slight, mild limitation of activity; the patient is comfortable at rest or with mild exertion	5	5	10
Class 3: Marked limitation of any activity; the patient is comfortable only at rest	3	3	6
Class 4: Any physical activity brings on discomfort and symptoms occur at rest	1	1	2
**Patient left ventricular ejection fraction**			
<40% EF (HFrEF)	3	4	7
>50% EF (HFpEF)	3	1	4
Between 40% and 50% EF	3	4	7
**Patient co-morbidity condition**			
Diabetes Type II	7	2	9
Hypertension	7	0	7
Chronic obstructive pulmonary disease	3	0	3
Depression	2	1	3
Osteoarthritis	0	3	3
Renal failure	2	0	2
Neuropathy	2	0	2
Obstructive sleep apnea	1	0	1
Anxiety	1	0	1
Gastro oesophageal reflux disease	1	0	1
Anemia	1	0	1
Bipolar	1	0	1
Dementia	1	0	1
Artrial fibrillation	1	0	1
Glaucoma	0	1	1
Obesity	0	1	1

^4^Patients classified NYHA Class 1 were excluded.

### Cognitive debriefing of the CBQ-HF

The results from the cognitive debriefing interviews indicate HF caregivers understood the final item wording and found the concepts included in the final version of the CBQ-HF relevant to their experiences as caregivers. Further detail on the number of caregivers who correctly interpreted each item and reported each item as relevant is included in Table 3. The changes made to version 1.0 of the CBQ-HF (as tested in set 1 interviews) following the interim analysis and changes made to version 2.0 of the CBQ-HF (as tested in set 2 interviews) are detailed below.

**Table 3:** Ease of understanding and relevance of the original 41 CBQ-HF v1.0 items

**Concept**	**Sub-concept**	**Item wording (‘During the past 4 week,…’)**	**Correct interpretation of item**	**No issue with item wording**	**Relevant to CHF caregiving experience**	**Example quote for relevance**
Physical burden	Tiredness	1…how much has caregiving made you feel physically tired?	17/18	17/18	14/18	“It’s so much to be done every day that I think I have been really tired” (04-06)
	Deterioration of own health (2 items)	**2…how much has caregiving caused you health problems?**	18/18	18/18	9/18	“because I have to be more active and on my feet with him, I have a little stress fracture in my foot.” (03-07)
		3…how much has caregiving made you neglect your own health	17/18	17/18	13/18	“doctor’s appointments and things like that, and I’ve been kind of pushing it back because of the time I’m doing this” (04-02)
	Physical effort	4…how much physical effort has it taken you to do caregiving tasks?*	13/18	13/18	9/18	“Somewhat, because I have to go physically, food shopping, cleaning up her place, doing things like that” (04-05)
	Lack of sleep	5…how much has caregiving made it difficult to sleep?	18/18	18/18	11/18	“I wake up in the middle of the night, I want to make sure that he’s OK” (03-05)
	Body aches	6…how much has caregiving made your body ache?*	12/18	17/18	8/18	“when I pick her up. And prop her up, and I’ll tweak my back a little bit” (03-02)
Emotional/ psychological burden	Overly depended upon (2 items)	7…how much have you felt like you need to do more for the person you care for?*	15/18	18/18	14/18	“I feel like I need to do more for her, but I really can’t.” (03-08)
		**8…how much have you felt overly relied upon by the person you care for**	18/18	18/18	12/18	“He asks a lot of me…he just don’t realize how much I do. And you feel like you don’t get credit” (04-09)
	Guilty (2 items)	9…how guilty have you felt because the time you spent caregiving limited what you can do for others?	15/18	17/18	12/18	“I wanted to take care of her [friend’s]dog while she’s in the hospital, but I couldn’t do that, and I can’t spend time with her as much as I would like to” (03-03)
		10…how guilty have you felt because you cannot do enough for the person you care for?	17/18	18/18	11/18	“Because I always feel a little guilty about not spending more time with her” (03-02)
	Frustrated	11…how much has caregiving made you feel frustrated?	18/18	18/18	14/18	“It’s very frustrating knowing you’ve given him good advice and he’s not following it.” (03-04)
	Stressed	12…how much has caregiving made you feel stressed?	15/18	17/18	14/18	“Every minute, every day. You know, there’s so much stress with medicines…I keep expecting to walk into the room and see him slumped over, and it’s just very stressful” (04-01)
	Resentment	**13…how much has caregiving made you feel resentful?**	15/18	15/18	6/18	“I resent when she gives me the guilties.” (03-06)
	Sad	14…how much has caregiving made you feel sad?	18/18	18/18	15/18	“feeling sad, make me cry, because I know that he might die” (03-03)
	Angry	*15…how much has caregiving made you feel angry?*	8/9	8/9	4/9	“He does make me mad at him when he doesn’t listen to what I say”(03-03)
	Depressed	**16…how much has caregiving made you feel depressed?**	16/18	17/18	11/18	“Definitely a lot. If I didn’t have to get out of bed for my four-year-old, I think maybe I’d stay in [bed]” (04-01)
	Inability to focus or concentrate	17…how much has caregiving made it difficult to concentrate on other things?*	16/18	17/18	12/18	“But when I’m constantly interrupted with phone calls during the work day, that's difficult for me” (03-06)
	Worry	18…how much has caregiving made you worry about the person you care for?	18/18	18/18	13/18	“I always worry about her a little. Because it’s easy for her to fall” (03-02)
	Mentally tired	19…how much has caregiving made you feel mentally tired?	17/18	17/18	13/18	“it does make me mentally tired when he challenges me” (03-03)
	Emotionally drained	20…how much has caregiving made you feel emotionally drained?	18/18	18/18	15/18	“there’s some draining in wanting to appease him while taking care of others (03-04)
	Overwhelmed	21…how much has caregiving made you feel overwhelmed?	17/18	17/18	12/18	“There are just times where I just get to the point where I can’t do this anymore” (03-07).
	Helpless	**22…how much has caregiving made you feel helpless?**	15/18	15/18	7/18	“it’s just like, now I can’t do this, you know? I just - I can’t help him.” (03-03)
	Inability to cope	*23…how much has caregiving made you feel like you can’t cope?*	8/9	8/9	6/9	“there are times where I had enough” (03-07)
	Isolation/ loneliness	24…how much has caregiving made you feel lonely?	18/18	18/18	9/18	“I don’t have any friends, basically.” (03-07)
	Support from others	25…how much support have you had from family or friends?	18/18	18/18	14/18	“my other family we all share and help with my grandmother” (04-04)
	Dislike caregiving (2 items)	*26…how uneasy have you felt while carrying out a personal care task for the person you care for (for example bathing or dressing them)?*	8/9	8/9	0/9	“He’s my husband. I don’t feel uneasy.” (03-04).
		27…how much have you enjoyed caregiving?	18/18	18/18	14/18	“I’m glad that I can provide that service to her” (03-02)
Social burden	Impact on relationship with patient	28…how much has caregiving caused problems in your relationship with the person you care for?	18/18	17/18	11/18	“he gets frustrated, and it causes arguments between the two of us(03-07).
	Impact on relationships with partner and family (2 items)	*29…how much has caregiving caused problems in your relationship with your partner or family?*	8/9	8/9	4/9	“And I don’t always discuss everything with her [daughter]…because I don’t want her to know how bad off her dad is. (03-03)
		30… how much has caregiving limited the time you spent with your partner or family?*	18/18	18/18	10/18	“there are times when I can’t do other things because I’m caring for her” (04-08)
	Impact on relationships with friends (2 items)	*31…how much has caregiving caused problems in your relationships with friends?*	8/9	8/9	4/9	“because of how limited I am with my time to spend with them” (03-04)
		**32…how much has caregiving limited the time you spent with friends?**	18/18	18/18	9/18	“we never go nowhere - with our friends no more. Never” (04-09)
	Impact on intimate relationships (2 items)	*33…how much has caregiving made it difficult to date?*	8/9	8/9	6/9	“I’m thinking of a single person that’s caregiving for someone else.” (03-04)
		*34…how much has caregiving caused problems with your sex life?*	9/9	9/9	7/9	“We have not had sex. He just haven’t felt like it.” (03-05).
Lifestyle burden	Lack of time for self (2 items)	35…how much have you felt like you have had enough time for yourself?*	18/18	18/18	15/18	“I feel like I have no down time” (04-05).
		*36…how much has caregiving limited time spent doing things for yourself? (for example going to the doctors for your own health)?*	9/9	8/9	5/9	“ I don’t do things for myself…because I put other people first ” (03-07)
	Lack of time to do non-caring tasks	**37…how much has caregiving made you feel unable to do the things you want to do?**	18/18	18/18	11/18	“It limits me from doing so many things that I would like to do, like travel.” (03-02).
	Avoid making plans or having to change plans	38…how much has caregiving caused you to change your plans or made you avoid making plans?	18/18	18/18	16/18	“A little, because we’re going to a wedding. And when he got tests coming up we’ll cancel.” (04-07)
	Unable to go on vacations or trips (2 items)	**39…how much has caregiving limited you travelling?**	17/18	17/18	10/18	“It’s my mother’s birthday this weekend, and I would have gone down for the whole weekend, But now I cannot do that.” (03-03)
		40…how much have you felt you cannot be away from the person you care for?	18/18	18/18	13/18	“She needs me and I need her. Just to check on each other, you know.” (03-08)
	Reduced working hours	41… how much has caregiving made it difficult to do paid work?	15/18	15/18	11/18	“I can only work part-time, and, uh, that’s even - some - at times difficult (03-07)

Key:

Item in *italicised* text: item deleted after first 9 interviews.

Item in **bold** text: item deleted after 18 interviews.

* Item wording modified after 18 interviews.

#### Deletion of items

Based on the analysis of the first set of cognitive interviews, eight items were deleted from version 1.0 of the questionnaire resulting in 33 items included in version 2.0 of the CBQ-HF. The rationale for the deletion of each item following the first set of cognitive interviews is presented in Table 4.

**Table 4:** Deletion of CBQ-HF items based on caregiver feedback in first set of cognitive interviews

**Items deleted following set 1 of cognitive debriefing interviews (n = 9 caregivers)**		
**Item**	**Number of caregivers who did not find the item relevant (or other rationale for deletion)**	**Example quote to support deletion**
How much has caregiving made you feel angry?	5/9 (plus conceptual overlap with the item dedicated on frustration)	*“I felt frustrated but not angry”* (03–02)
How much has caregiving made you feel like you can’t cope?	6/9 (plus conceptual overlap with the item ‘*how much has caregiving made you feel helpless*’)	*“I’m used to all -this. It’s nothing new, you know?”* (03–08)
How uneasy have you felt while carrying out a personal care task for the person you care?	9/9	“*I’m not feeling uneasy, because that’s something that I would do for him anyways*” (03–05)
How much has caregiving caused problems in your relationship with your partner and family?	5/9 (plus conceptual overlap with item on the amount of time spent with the patient)	*“I don’t know how to answer that. It’s like part of all the other questions,”* (03–08)
How much has caregiving caused problems in your relationships with friends?	6/9 (plus caregivers found the item too broad)	*“My friends are very understanding. So I would say not at all. I think that they understand where I'm at.”* (03–06)
How much has caregiving made it difficult to date?	6/9	*“I did not date for other reasons, because I’m married - so I don’t go on dates.”* (03–02)
How much has caregiving caused problems with your sex life?	5/9 (reported sex life difficulties a result of other illnesses, work, partner’s mood)	“*Because there really isn’t any. Due to his medication, due to his back problems. That makes it very difficult for him*.” (03–07)
How much has caregiving limited time spent doing things for yourself?	4/9 (plus conceptual overlap with ‘*how much have you felt like you had enough time for yourself*?’ and ‘*how much has caregiving made you feel unable to do the things you want to do?*’.	“*not at all because I am able to do the things - may not just be when I want to do it*” (04–08)

Following the second set of cognitive interviews, a further seven items were deleted, leading to a 26-item version of the CHQ-HF referred to as version 3.0 (Additional file 1). The rationale for item reduction was primarily based on whether the item was relevant to caregivers or lacked clarity but also to achieve a manageable number of items for ease-of-use and respondent acceptability of the CBQ-HF. The rationale for the deletion of each item following the second set of cognitive interviews is presented in Table 5. In each case, the rationale for deletion is supported by an example quotation from a caregiver, highlighting the main reason for deletion of the item.

**Table 5:** Deletion of CBQ-HF items based on caregiver feedback in second set of cognitive interviews

**Items deleted following set 2 cognitive debriefing interviews based on feedback from all 18 caregivers**		
**Item**	**Number of caregivers who did not find the item relevant (or other rationale for deletion)**	**Example quote to support deletion**
How much has caregiving caused you health problems?	8/18 (plus caregivers found it difficult to attribute health problems specifically to caregiving)	“*Not at all. I haven’t had any problems related - not that I know of*” (04–08)
How much have you felt overly relied upon by the person you care for?	6/18 (plus conceptual equivalence to the item ‘*have you felt the person you care for asks for too much’* considered easier to understand)	“*I would say we’ve been through this before. You know the rules*” (03–08)
How much has caregiving made you feel resentful?	12/18 (plus 3/18 caregivers found it difficult to understand)	*“Not at all. I don’t resent that, because my grandfather took care of me as a kid”* (04–03)
How much has caregiving made you feel depressed?	7/18 (plus 4/9 caregivers from set 1 considered ‘depression’ too severe or clinical a term; 15/18 caregivers found the item ‘*how much has caregiving made you feel sad’* more relevant	*“I don’t think both of those are necessary because it’s kind of the same. I prefer sad just because depressed just sounds…a little harsher, I guess”* (04–04)
How much has caregiving made you feel helpless?	11/18 (plus 12/18 caregivers considered the item ‘*has caregiving made you feel overwhelmed’* more relevant)	***“****But I can do it, help her with things, so [it isn’t relevant]”* (03–08)
How much have you been unable to do the things you want to do?	7/18 (plus 15/18 caregivers considered the item ‘*have you felt like you have no time for yourself’* more relevant)	*“There’s nothing I really want to do right now.”* (03–04)
How much has caregiving limited your travelling?	8/18 (plus conceptual equivalence with the items ‘*has caregiving caused you to change/avoid making plans’* and ‘*have you felt you cannot be away from the person you care for*’ considered more relevant.)	*“Because I drive, I do what I want to do - as far as traveling.”* (03–05)

#### Item modifications

To further optimise the clarity and relevance of the questionnaire content, a number of items were modified following the first and second set of cognitive interviews. The rationale for changes is detailed in Table 6.

**Table 6:** Item optimization

**Items modified following cognitive debriefing interviews (N = 18 caregivers)**		
**Item/sub-domain**	**Rationale for modification following set 1 (n = 9)**	**Rationale for modification following set 2 (n = 9)**
Physical effort	Item wording changed from ‘*how much physical effort has it taken you to do caregiving tasks’* to ‘*how much has caregiving been physically hard work*?’ to improve clarity and understanding to caregivers.	No further modifications
Body ache	3/9 caregivers thought about pain as well as aches so item changed to ‘*During the past 4 weeks, how much has caregiving caused you physical aches and pains?*’	The term ‘physical’ was removed as caregivers felt it was redundant. Final item wording: ‘*how much has caregiving caused you aches and pains*?’
Feeling overly depended upon	Item revised to use a frequency scale rather than an intensity scale to fit better with the item wording. Revised item wording: *‘how much of the time have you felt you need to do more for the person you care for?*’	More than half of the caregivers preferred to omit the new wording *how much of the time’*. Item returned to original wording: ‘*how much have you felt like you need to do more for the person you care for*?’
Difficulty concentrating/focusing	The term ‘focus’ was included alongside ‘concentrate’ to improve clarity and interpretation. Revised item wording: *‘how much has caregiving made it difficult to focus or concentrate on other things?*’	Including ‘focus’ alongside ‘concentrate’ appeared to help most caregivers interpret the item correctly. No further item modifications.
Lack of time for self	To be consistent with the negative phrasing of the other items and to avoid confusion, the item was changed from ‘*how much have you felt like you have had enough time for yourself*?’ to ‘*how much have you felt like you have no time for yourself?*’	No further item modifications.
Time spent with family and partner time spent with friends	Two separate items were tested for these two sub-domains and feedback demonstrated that caregivers found both items easy to understand and relevant.	In the final item the two separate items were merged to reduce the number of items overall. Final item wording: ‘*how much has caregiving limited the time you spent with partner, family or friends?*’

#### Changes to wording of instructions

The majority of caregivers (15/18) understood the questionnaire instructions without any difficulty. While seventeen caregivers did not report any problems with the term ‘chronic heart failure’, the main problem reported was with the use of the acronym ‘CHF’ with one caregiver interpreting it as “congestive heart failure”. For this reason it was felt that the briefer term ‘heart failure’ throughout the questionnaire would ensure consistent understanding in version 3.0 of the CBQ-HF.

#### Response options and scale

The majority of caregivers understood and used the response options appropriately when completing the questionnaire during the ‘think-aloud’ exercise. In order to evaluate caregivers’ preference for a severity versus a frequency response scale, both versions were tested in the interviews. A severity response scale (from ‘Not at all’ to ‘A lot’) was included in the actual questionnaire that caregivers completed and a frequency response scale (from ‘Never’ to ‘Nearly Always’) was presented on a showcard at the end of the interview. Of those that were asked (13/18) slightly more caregivers (6/13) preferred the severity response scale: *“I think this more like reflects really the reality of it”* (male caregiver aged 42) and the remainder did not have a preference for either version. In light of the caregiver’s overall favourable feedback for the intensity response scale, the developers chose to retain it in version 3.0 of the CBQ-HF.

#### Recall period

The pre-specified recall period of 4-weeks was well-understood by all but one caregiver when answering the questionnaire. The one caregiver who had difficulty reported that thinking back over a longer period such as months or years was preferred. Six caregivers suggested having a longer recall period predominantly because this would allow them to report a greater number and/or severity of burdens. That said, all six used the stated 4-week recall period and did not comment that this posed any problem when answering the questionnaire as part of the ‘think-aloud’ exercise. Therefore, the 4-week recall period was retained in version 3.0 of the CBQ-HF.

## Discussion

The primary objective of this research was to evaluate the face and content validity of a newly developed caregiver burden questionnaire through cognitive interviews, as advocated by regulators and experts in COA development [18,19]. Cognitive testing of version 1.0 of CBQ-HF and subsequent item reduction and modification in version 2.0 has led to a shortened version 3.0 of the CBQ-HF. Version 3.0 of the CBQ-HF includes 26 items, measured on a 5-point Likert severity scale, assessing 1) Physical Burdens (5 items); 2) Emotional/Psychological Burdens (15 items); 3) Social Burdens (2 items); and 4) Lifestyle Burdens (4 items). Eight items were deleted from version 1.0 following the first set of cognitive interviews and a further seven items were deleted from version 2.0 following the second set of cognitive interviews. Definitions were modified, as was the wording of an additional six items in version 2.0. Decisions to delete and modify the questionnaire content were made with expert input throughout the process to ensure that the most important conceptual domains were retained and the item wording remained conceptually clear and easy to understand for caregivers. The inclusion of caregivers with a range of educational backgrounds both in the initial development of the conceptual model [25] and during this validation research has ensured that the CBQ-HF is understandable to caregivers with varying education and health literacy levels by using caregiver-friendly language.

Caregivers undertake a whole range of activities to assist with the daily living of HF patients. Clark and colleagues comment that it is important to consider both the invisible (e.g. monitoring signs of symptom exacerbation or risk) and visible care activities (e.g. dressing, medication management) performed by caregivers of HF patients [17]. It has been estimated that many of HF hospitalisations are preventable, with poor adherence to medical regimes and failure to seek help for escalating symptoms cited as the most common reasons [5]. Caregivers have a key role to play in these activities as they span both visible (e.g. medication management, dressing, bathing and help-seeking) and invisible care activities (e.g. monitoring signs of symptom exacerbation) [17]. The CBQ-HF would be valuable in helping to understand and monitor the relationship between patient HF severity and caregiver burden resulting from these caregiving activities. Tracking the relationship between caregiver burden and patient service use could help in understanding the wider context of health care systems and in particular the integration of HF caregivers into the disease management [5,36,37], as well as placing an economic value on the work of caregivers [38]. The CBQ-HF could also be valuable in evaluating caregiver outcomes of interventions targeting the patient or caregiver alone or the patient-caregiver dyad [5,39]. It may also be used in clinical practice to assess caregiver’s need for support and could also offer a structured way for clinicians and social care professionals to discuss experiences of caregiver burden across a number of domains of life.

One of the limitations of the CBQ-HF is that it only assesses the negative aspects of caregiving. In a number of studies, it has been reported that caregivers can feel enriched due to caring for a loved one [40,41]. However, during the initial development of the CBQ-HF item pool, it was agreed that measuring the positive aspects of caring was not the current objective for this instrument. Nevertheless, some positive impacts were reported during the early concept elicitation work [29] but these were reported by a minority of caregivers. Further qualitative research could therefore be warranted to understand the positive consequences of caregiving and if relevant, additional items that capture these domains could be included into the CBQ-HF to ensure it captures the whole spectrum of caregiver experiences.

Furthermore, it should be noted that the CBQ-HF is designed to assess subjective caregiver burden. It does not assess or capture objective caregiver burden (such as the number of actual tasks completed). To fully understand the caregiver experience, it would be valuable to use both approaches. We would therefore support the combined use of the CBQ-HF v3.0 alongside an objective measure (such as the DOBI) [21,22] to comprehensively assess caregiver burden. Further research may wish to explore the feasibility and appropriateness of using both of these questionnaires within a clinical trial setting.

In addition, further work is planned to assess the psychometric properties and cross-cultural appropriateness of version 3.0 of the CBQ-HF. This study has provided a thorough assessment of content validity in a US-English sample. A Translatability Assessment^SM^ of the measure has since been performed confirming the cultural appropriateness of the measure so that it may be used in ex-US countries.

The next step in the development of the CBQ-HF will be to assess the reliability and validity of the new questionnaire in a longitudinal study with a much larger sample of caregivers. This larger study may also examine the association between the severity of patient’s HF and caregiver burden measured by the CBQ-HF to evaluate the CBQ-HF’s discriminative validity and sensitivity to change. A quantitative analysis of the item responses between patient’s HF severity (and other patient/caregiver subgroups) was not conducted during this study as it would most likely lack integrity and robustness due to the small sample size. While analyzing the data for the effect of HF severity on caregiver burden is important and of interest, we feel that this would be better suited to future validation studies conducted with a larger sample.

The objective of this study was to develop and assess the conceptual relevance of the CBQ-HF specifically for HF caregivers. The CBQ-HF items were developed following qualitative interviews with HF caregivers which supports the content validity of the CBQ-HF for use with caregivers of HF patients. Nevertheless, the concepts measured and the non-specific nature of the item wording is likely to be relevant to caregivers of patients with other chronic diseases. The CBQ-HF could therefore be adapted for use in other chronic conditions e.g. diabetes, myocardial infarction, arrhythmias, COPD and also possibly in some types of less advanced cancer. In adapting the measure for other illness areas, a number of the original items could be retained as the ‘core’ questionnaire with supplementary questions designed (and appropriately validated) for other specific conditions.

## Conclusion

In conclusion, the CBQ-HF is a comprehensive and relevant measure of subjective caregiver burden in HF. This study has established that it has strong face and content validity and should be valuable as both an outcomes measure and possibly as a tool for clinical practice. The cultural appropriateness of the CBQ-HF has since been established and a future study is planned to evaluate the psychometric properties of the new measure.

## Abbreviations

CBQ-HF: Caregiver Burden Questionnaire for Heart Failure; COA: Clinical outcome assessment; EF: Ejection fraction; FDA: Food and drugs administration; HF: Heart failure; HFrEF: Heart failure reduced ejection fraction; HFpEF: Heart failure preserved ejection fraction; N: Sample number; NYHA: New York Heart Association; SD: Standard deviation

## Competing interests

Mr Blackburn, Ms Maguire and Ms Humphrey were contracted by Novartis as consultants to perform the study and develop the manuscript.

## Authors’ contributions

LH, SB and LM conceived and developed the study design, carried out the acquisition of data, analysis and interpretation of data, and drafted the manuscript. KK, CD and AS have helped to draft the manuscript and revise it critically for important intellectual content. All authors read and approved the final manuscript.

## Supplementary Material

Additional file 1 Caregiver Burden Questionnaire - Heart Failure Version 3.0 (CBQ-HF). Click here for file
