# Supplementary material for: The Caregiver Burden Questionnaire for Heart Failure (CBQ-HF): face and content validity
Source: Health Qual Life Outcomes. 2013 May 25;11:84. doi: 10.1186/1477-7525-11-84 (PMC3673843; doi:10.1186/1477-7525-11-84)
Supplement: Additional file 1 — Caregiver Burden Questionnaire - Heart Failure Version 3.0 (CBQ-HF). [file 1477-7525-11-84-S1.docx]

**Caregiver Burden Questionnaire - Heart Failure Version 3.0**

**(CBQ-HF)**

The following questions ask you about what it is like caregiving for someone with heart failure.

By ‘Caregiving’ we mean all the things you do to help a relative or friend with heart failure, for which you are not paid.

If that person has other health conditions, please try to think about their heart failure only and not any other health problems.

For each question, think about how caregiving for a person with heart failure has affected you during the **past 4 weeks**.

Check only **one box** for each question that best reflects how you feel.

**Section 1: Your Physical Well-Being**

| 1. During the past 4 weeks, how much has caregiving made you feel **physically tired**? | Not at all  🞎 | A little  🞎 | | Somewhat  🞎 | Quite a lot  🞎 | A lot  🞎 |
| --- | --- | --- | --- | --- | --- | --- |
| 1. During the past 4 weeks, how much has caregiving made you **neglect** your **own health**? | Not at all  🞎 | A little  🞎 | | Somewhat  🞎 | Quite a lot  🞎 | A lot  🞎 |
| 1. During the past 4 weeks, how much has caregiving been **physically hard work?** | None  🞎 | A little  🞎 | | Somewhat  🞎 | Quite a lot  🞎 | A lot  🞎 |
| 1. During the past 4 weeks, how much has caregiving made it **difficult to sleep**? | Not at all  🞎 | A little  🞎 | | Somewhat  🞎 | Quite a lot  🞎 | A lot  🞎 |
| 1. During the past 4 weeks, how much has caregiving caused you **aches and pains**? | Not at all  🞎 | | A little  🞎 | Somewhat  🞎 | Quite a lot  🞎 | A lot  🞎 |

**Section 2: Your Emotional Well-Being**

| 1. During the past 4 weeks, how much have you felt like **you need to do more** for the person you care for? | Not at all  🞎 | A little  🞎 | Somewhat  🞎 | Quite a lot  🞎 | A lot  🞎 |
| --- | --- | --- | --- | --- | --- |
| 1. During the past 4 weeks, how much have you felt the person you care for **asks too much** of you? | Not at all  🞎 | A little  🞎 | Somewhat  🞎 | Quite a lot  🞎 | A lot  🞎 |
| 1. During the past 4 weeks, how **guilty** have you felt because the time you spent caregiving **limited** what you can **do for others**? | Not at all  🞎 | A little  🞎 | Somewhat  🞎 | Quite a lot  🞎 | A lot  🞎 |
| 1. During the past 4 weeks, how **guilty** have you felt because you **cannot do enough** for the person you care for? | Not at all  🞎 | A little  🞎 | Somewhat  🞎 | Quite a lot  🞎 | A lot  🞎 |
| 1. During the past 4 weeks, how much has caregiving made you feel **frustrated**? | Not at all  🞎 | A little  🞎 | Somewhat  🞎 | Quite a lot  🞎 | A lot  🞎 |
| 1. During the past 4 weeks, how much has caregiving made you feel **stressed**? | Not at all  🞎 | A little  🞎 | Somewhat  🞎 | Quite a lot  🞎 | A lot  🞎 |
| 1. During the past 4 weeks, how much has caregiving made you feel **sad**? | Not at all  🞎 | A little  🞎 | Somewhat  🞎 | Quite a lot  🞎 | A lot  🞎 |
| 1. During the past 4 weeks, how much has caregiving made it **difficult to focus or concentrate on other things**? | Not at all  🞎 | A little  🞎 | Somewhat  🞎 | Quite a lot  🞎 | A lot  🞎 |
| 1. During the past 4 weeks, how much has caregiving made you **worry** about the person you care for? | Not at all  🞎 | A little  🞎 | Somewhat  🞎 | Quite a lot  🞎 | A lot  🞎 |
| 1. During the past 4 weeks, how much has caregiving made you feel **mentally tired**? | Not at all  🞎 | A little  🞎 | Somewhat  🞎 | Quite a lot  🞎 | A lot  🞎 |
| 1. During the past 4 weeks, how much has caregiving made you feel **emotionally drained**? | Not at all  🞎 | A little  🞎 | Somewhat  🞎 | Quite a lot  🞎 | A lot  🞎 |
| 1. During the past 4 weeks, how much has caregiving made you feel **overwhelmed**? | Not at all  🞎 | A little  🞎 | Somewhat  🞎 | Quite a lot  🞎 | A lot  🞎 |
| 1. During the past 4 weeks, how much has caregiving made you feel **lonely**? | Not at all  🞎 | A little  🞎 | Somewhat  🞎 | Quite a lot  🞎 | A lot  🞎 |
| 1. During the past 4 weeks, how much **support** have you had **from family or friends**? | Not at all  🞎 | A little  🞎 | Somewhat  🞎 | Quite a lot  🞎 | A lot  🞎 |
| 1. During the past 4 weeks, how much have you **enjoyed** caregiving? | Not at all  🞎 | A little  🞎 | Somewhat  🞎 | Quite a lot  🞎 | A lot  🞎 |

**Section 3: Your Social Life & Relationships**

| 1. During the past 4 weeks, how much has caregiving caused problems in your relationship with the **person you care for?** | Not at all  🞎 | A little  🞎 | Somewhat  🞎 | Quite a lot  🞎 | A lot  🞎 |
| --- | --- | --- | --- | --- | --- |
| 1. During the past 4 weeks, how much has caregiving **limited the time** you spent with **partner, family or friends**? | Not at all  🞎 | A little  🞎 | Somewhat  🞎 | Quite a lot  🞎 | A lot  🞎 |

**Section 4: Your Lifestyle**

| 1. During the past 4 weeks, how much have you felt like you have **no time for yourself**? | Not at all  🞎 | A little  🞎 | Somewhat  🞎 | Quite a lot  🞎 | A lot  🞎 |
| --- | --- | --- | --- | --- | --- |
| 1. During the past 4 weeks, how much has caregiving caused you to **change your plans or made you avoid making plans**? | Not at all  🞎 | A little  🞎 | Somewhat  🞎 | Quite a lot  🞎 | A lot  🞎 |
| 1. During the past 4 weeks, how much have you felt **you cannot be away from the person you care for**? | Not at all  🞎 | A little  🞎 | Somewhat  🞎 | Quite a lot  🞎 | A lot  🞎 |
| 1. During the past 4 weeks, how much has caregiving made it **difficult** to do paid **work**? | Not at all  🞎 | A little  🞎 | Somewhat  🞎 | Quite a lot  🞎 | A lot  🞎 |
